# Supplementary material for: Use of machine learning to identify protective factors for death from COVID-19 in the ICU: a retrospective study
Source: PeerJ. 2024 Jun 12;12:e17428. doi: 10.7717/peerj.17428 (PMC11179634; doi:10.7717/peerj.17428)
Supplement: Supplemental Information 2 [file peerj-12-17428-s002.pdf]

Use of machine learning to identify protective factors for death from COVID-19 in the ICU

Lander Dos Santos1 , Lincoln Luis Silva2 , Fernando Castilho Pelloso3 , Vinicius Maia4 , Constanza Pujals1 , Deise Helena Pelloso Borghesan5 , Maria Dalva Carvalho1 , Raíssa Bocchi Pedroso1 , Sandra Marisa Pelloso1 .

[1] Graduate Program in Health Sciences, State University of Maringá, Maringá, Paraná, Brazil

[2] Department of Emergency Medicine, Duke University School of Medicine, Durham, North Carolina, United States of America

[3] Department of Medicine, Federal University of Paraná, Curitiba, Paraná, Brazil

[4] UniCesumar, Maringá, Paraná, Brazil

[5] Union of Catholic Colleges of Mato Grosso, Cuiabá, Mato Grosso, Brazil

Corresponding Author:
Professor Sandra Marisa Pelloso

Av. Colombo, 5,790 - Bloco 126, Maringá, Paraná, 87030-230, Brazil
Email address: smpeloso@gmail.com

Variables classified as nearly zero variance were removed, meaning they were not used in the Random Forest and Logistic Regression models.

Table 1. Zero and almost zero variance

Variable	Frequency ratio	Unique values (%)	Variance null	Almost zero variance
Age	1.000000	10.2209945	FALSE	FALSE
Sex	1.610108	0.4143646	FALSE	FALSE
Weight	1.631579	4.4198895	FALSE	FALSE
Height cm	1.057143	2.6243094	FALSE	FALSE
BMI	1.000000	9.8066298	FALSE	FALSE
Unit Outcome	1.733840	0.2762431	FALSE	FALSE
Decision on palliative care	119.000000	0.4143646	FALSE	TRUE
Length of stay in ICU	1.022222	6.3535912	FALSE	FALSE
Length of hospital stay	1.131579	8.4254144	FALSE	FALSE
NYHA Classification 2 or 3	10.040816	0.2762431	FALSE	FALSE

Table 1. Zero and almost zero variance

Variable	Frequency ratio	Unique values (%)	Variance null	Variance almost zero
Non-dialysis chronic renal failure	18.321429	0.2762431	FALSE	FALSE
Dialysis Chronic Renal Failure	269.500000	0.2762431	FALSE	TRUE
Cirrhosis Child A or B	179.333333	0.2762431	FALSE	TRUE
Cirrhosis Child C	540,000000	0.2762431	FALSE	TRUE
Liver Failure	134.250000	0.2762431	FALSE	TRUE
Locoregional solid tumor	53.100000	0.2762431	FALSE	TRUE
Metastatic Solid Tumor	540,000000	0.2762431	FALSE	TRUE
Tumor site	2,000000	0.8287293	FALSE	FALSE
Malignant hematological disease	107.200000	0.2762431	FALSE	TRUE
Type of malignant hematological disease	3,000000	0.4143646	FALSE	FALSE
Name of malignant hematological disease	3,000000	0.4143646	FALSE	FALSE
Immunosuppression	48.181818	0.2762431	FALSE	TRUE
severe COPD	13.236842	0.2762431	FALSE	FALSE
Use of Steoids	269.500000	0.2762431	FALSE	TRUE
AIDS	269.500000	0.2762431	FALSE	TRUE
Systemic Arterial Hypertension	2.339506	0.2762431	FALSE	FALSE
Asthma	26.050000	0.2762431	FALSE	TRUE
Uncomplicated diabetes	2.005556	0.2762431	FALSE	FALSE
Complicated diabetes	12.195122	0.2762431	FALSE	FALSE
Angina	269.500000	0.2762431	FALSE	TRUE
Previous Acute Myocardial Infarction	32.812500	0.2762431	FALSE	TRUE
Arrhythmia	66.625000	0.2762431	FALSE	TRUE
Peripheral Artery Disease	179.333333	0.2762431	FALSE	TRUE

Table 1. Zero and almost zero variance

Variable	Frequency ratio	Unique values (%)	Variance null	Variance almost zero
Chronic arterial fibrillation	40.615385	0.2762431	FALSE	TRUE
Rheumatic Disease	107.200000	0.2762431	FALSE	TRUE
Stroke sequelae	29.055556	0.2762431	FALSE	TRUE
Stroke without sequelae	107.200000	0.2762431	FALSE	TRUE
Insanity	26.050000	0.2762431	FALSE	TRUE
Smoking	21.541667	0.2762431	FALSE	TRUE
Alcoholism	53.100000	0.2762431	FALSE	TRUE
Psychiatric Illness	9.018519	0.2762431	FALSE	FALSE
Morbid obesity	3.918182	0.2762431	FALSE	FALSE
Malnutrition	89.166667	0.2762431	FALSE	TRUE
Solid Organ Transplant	269.500000	0.2762431	FALSE	TRUE
Autologous blood transfusion	540,000000	0.2762431	FALSE	TRUE
Intestine Transplant	134.250000	0.2762431	FALSE	TRUE
Lung transplant	540,000000	0.2762431	FALSE	TRUE
kidney transplant	134.250000	0.2762431	FALSE	TRUE
Hypothyroidism	9.018519	0.2762431	FALSE	FALSE
Hyperthyroidism	540,000000	0.2762431	FALSE	TRUE
Dyslipidemia	32.812500	0.2762431	FALSE	TRUE
Chemotherapy	179.333333	0.2762431	FALSE	TRUE
Radiotherapy	269.500000	0.2762431	FALSE	TRUE
History of Pneumonia	134.250000	0.2762431	FALSE	TRUE
Delirium Obnubilation Stupor or With the	4.658730	0.2762431	FALSE	FALSE
Seizures or Epilepsy	355.500000	0.2762431	FALSE	TRUE
Focal neurological deficit	355.500000	0.2762431	FALSE	TRUE

Table 1. Zero and almost zero variance

**Variable**	**Frequency ratio**	**Unique values (%)**	**Variance null**	**Variance almost zero**
Hypovolemic or hemorrhagic shock	712,000000	0.2762431	FALSE	TRUE
Septic shock	70.300000	0.2762431	FALSE	TRUE
Heart Rhythm Disorders	78.222222	0.2762431	FALSE	TRUE
Anaphylactic or undefined shock	712,000000	0.2762431	FALSE	TRUE
Neurosurgery	712,000000	0.2762431	FALSE	TRUE
Respiratory failure in the first hour	4.974790	0.2762431	FALSE	FALSE
Mechanical Ventilation in the First Hour	1.402027	0.2762431	FALSE	FALSE
Non-invasive ventilation in the first hour	2.276498	0.2762431	FALSE	FALSE
Vasopressors in the first hour	wha1.594891	0.2762431	FALSE	FALSE
Cardiac arrhythmias in the first hour	78.000000	0.2762431	FALSE	TRUE
Cardiorespiratory arrest in the first hour	63.636364	0.2762431	FALSE	TRUE
Acute kidney injury in the first hour	100.571429	0.2762431	FALSE	TRUE
Hemodialysis in the first hour	78.000000	0.2762431	FALSE	TRUE
Asystole in the first hour	176.750000	0.2762431	FALSE	TRUE
Pulseless electrical activity in the first hour	100.571429	0.2762431	FALSE	TRUE
Atrial fibrillation in the first hour	176.750000	0.2762431	FALSE	TRUE
Sustained ventricular tachycardia in the first hour	710,000000	0.2762431	FALSE	TRUE
Acute respiratory failure in the first hour	1.672932	0.2762431	FALSE	FALSE
Mechanical ventilation	1.821429	0.2762431	FALSE	FALSE
Non-invasive ventilation	1.672932	0.2762431	FALSE	FALSE

Table 1. Zero and almost zero variance

Variable	Frequency ratio	Unique values (%)	Variance null	Variance almost zero
Vasopressors	1.410169	0.2762431	FALSE	FALSE
Arrhythmias	70.100000	0.2762431	FALSE	TRUE
Cardiorespiratory Arrest	38.500000	0.2762431	FALSE	TRUE
Acute Kidney Injury	100.571429	0.2762431	FALSE	TRUE
Hemodialysis	63.636364	0.2762431	FALSE	TRUE
Asystole	58.250000	0.2762431	FALSE	TRUE
Pulseless electrical activity	78.000000	0.2762431	FALSE	TRUE
Atrial fibrillation	176.750000	0.2762431	FALSE	TRUE
Sustained ventricular tachycardia	354.500000	0.2762431	FALSE	TRUE
Lowest SBP 1h	1.071429	16.5745856	FALSE	FALSE
Lowest PAD 1h	1.000000	12.1546961	FALSE	FALSE
Lowest MAP 1h	1.000000	29.8342541	FALSE	FALSE
Highest HR 1h	1.277778	13.6740331	FALSE	FALSE
Highest FR 1h	2.206349	5.2486188	FALSE	FALSE
Highest Temperature 1h	2.581395	8.0110497	FALSE	FALSE
Minor Glasgow Coma Scale 1h	2.035088	1.2430939	FALSE	FALSE
Higher leukocyte count 1h	1.000000	42.5414365	FALSE	FALSE
Lower Platelet Count 1h	1.000000	44.8895028	FALSE	FALSE
Highest creatinine 1h	1.000000	7.5966851	FALSE	FALSE
Higher Bilirubin 1h	1.142857	2.3480663	FALSE	FALSE
Higher PH 1h	1.236842	8.5635359	FALSE	FALSE
Lower PH1h	1.000000	6.7679558	FALSE	FALSE
Highest PaO2 1h	1.000000	19.0607735	FALSE	FALSE
Lowest PaO2 1h	1.428571	10.6353591	FALSE	FALSE
Higher PaCO21h	1.250000	11.6022099	FALSE	FALSE

Table 1. Zero and almost zero variance

Variable	Frequency ratio	Unique values (%)	Variance null	Variance almost zero
Lowest PaCO2 1h	1.181818	7.1823204	FALSE	FALSE
Highest Fi O21h	1.341085	4.0055249	FALSE	FALSE
Lowest Fi O21h	1.045455	4.8342541	FALSE	FALSE
Higher PaO2Fi O21h Ratio	1.000000	43.9226519	FALSE	FALSE
Lowest PaO2FiO2 Ratio 1h	1.000000	19.8895028	FALSE	FALSE
Highest Lactate 1h	1.727273	4.2817680	FALSE	FALSE
Urea	1.058824	21.9613260	FALSE	FALSE
BUN	1.058824	21.9613260	FALSE	FALSE
Non-invasive mechanical ventilation	1.559140	0.2762431	FALSE	FALSE
Failure of non-invasive ventilation	4.368421	0.2762431	FALSE	FALSE
Mechanical ventilation	5.318584	0.2762431	FALSE	FALSE
Duration of mechanical ventilation	1.000000	5.9392265	FALSE	FALSE
Tracheostomy	4.028169	0.2762431	FALSE	FALSE
High flow mask	356,000000	0.2762431	FALSE	TRUE
Duration of Hemodialysis	1.117647	3.5911602	FALSE	FALSE
Extended Hemodialysis in Acute Kidney Injury	129.000000	0.2762431	FALSE	TRUE
Ischemic Heart Disease	129.000000	0.2762431	FALSE	TRUE
Central Venous Catheter	5.672897	0.2762431	FALSE	FALSE
Indwelling bladder catheter	7.022472	0.2762431	FALSE	FALSE
PAM catheter	2.718750	0.2762431	FALSE	FALSE
Intraaortic balloon	713.000000	0.2762431	FALSE	TRUE
Minimally invasive hemodynamic monitoring	118,000000	0.2762431	FALSE	TRUE
Transfusion	9.500000	0.2762431	FALSE	FALSE
Red blood cell concentrate	12.730769	0.2762431	FALSE	FALSE

**Table 1.** Zero and almost zero variance				
**Variable**	**Frequency ratio**	**Unique values (%)**	**Variance null**	**Variance almost zero**
Fresh Frozen Plasma	58.500000	0.2762431	FALSE	TRUE
Thrombolytic Agents	356,000000	0.2762431	FALSE	TRUE

In Table 2, we present the missing values per variable. Note that some variables have more than 99% missing values. All variables with more than 50% missing values were eliminated.

**Table 2.** Missing values per variable	
**Variable**	**Amount**
Type of malignant hematological disease	719 (99.31%)
Name of malignant hematological disease	719 (99.31%)
Tumor site	713 (98.48%)
Higher Bilirubin 1h	644 (88.95%)
Highest Lactate 1h	601 (83.01%)
Duration of Hemodialysis	594 (82.04%)
Height cm	565 (78.04%)
BMI	565 (78.04%)
Weight	561 (77.49%)
Lowest Fi O21h	540 (74.59%)
Lowest PaO2FiO2 Ratio 1h	540 (74.59%)
Lower PH1h	533 (73.62%)
Lowest PaO2 1h	533 (73.62%)
Lowest PaCO2 1h	533 (73.62%)
Minor Glasgow Coma Scale 1h	516 (71.27%)
NYHA Classification 2 or 3	183 (25.28%)
Non-dialysis chronic renal failure	183 (25.28%)

Table 2. Missing values per variable

Variable	Amount
severe COPD	183 (25.28%)
Systemic Arterial Hypertension	183 (25.28%)
Uncomplicated diabetes	183 (25.28%)
Complicated diabetes	183 (25.28%)
Psychiatric Illness	183 (25.28%)
Morbid obesity	183 (25.28%)
Hypothyroidism	183 (25.28%)
Higher PaO2Fi O21h Ratio	161 (22.24%)
Highest Fi O21h	159 (21.96%)
Duration of mechanical ventilation	123 (16.99%)
Urea	84 (11.6%)
BUN	84 (11.6%)
Lower Platelet Count 1h	74 (10.22%)
Highest creatinine 1h	73 (10.08%)
Higher PH 1h	72 (9.94%)
Highest PaO2 1h	71 (9.81%)
Higher PaCO21h	71 (9.81%)
Higher leukocyte count 1h	70 (9.67%)
Lowest SBP 1h	34 (4.7%)
Lowest PAD 1h	34 (4.7%)
Lowest MAP 1h	34 (4.7%)
Highest FR 1h	34 (4.7%)
Highest HR 1h	33 (4.56%)
Highest Temperature 1h	33 (4.56%)
Respiratory failure in the first hour	13 (1.8%)
Mechanical Ventilation in the First Hour	13 (1.8%)

Table 2. Missing values per variable

Variable	Amount
Non-invasive ventilation in the first hour	13 (1.8%)
Vasopressors in the first hour	13 (1.8%)
Acute respiratory failure in the first hour	13 (1.8%)
Mechanical ventilation	13 (1.8%)
Non-invasive ventilation	13 (1.8%)
Vasopressors	13 (1.8%)
Delirium Obnubilation Stupor or Coma	11 (1.52%)
Non-invasive mechanical ventilation	10 (1.38%)
Failure of non-invasive ventilation	10 (1.38%)
Mechanical ventilation	10 (1.38%)
Tracheostomy	10 (1.38%)
Central Venous Catheter	10 (1.38%)
Indwelling bladder catheter	10 (1.38%)
PAM catheter	10 (1.38%)
Transfusion	10 (1.38%)
Red blood cell concentrate	10 (1.38%)
Unit Outcome	5 (0.69%)
Age	0 (0%)
Sex	0 (0%)
Length of stay in ICU	0 (0%)
Length of hospital stay	0 (0%)

Table 3 presents the information gain per variable. The variables that stand out the most are the use and duration of mechanical ventilation. Additionally, approximately 37% of the predictor variables show no information gain. Those with null information gain were eliminated

Table 3. Information gain per variable

Variable	Information gain
Duration of mechanical ventilation	127.768816278
Mechanical ventilation	111.471292165
Central Venous Catheter	62.573210305
Indwelling bladder catheter	55.673934628
Length of hospital stay	43.551346100
PAM catheter	28.408457968
Urea	27.628248354
BUN	27.628248354
Mechanical ventilation	22.191547442
Age	20.336069126
Vasopressors	19.573846271
Highest creatinine 1h	18.157034301
Vasopressors in the first hour	14.328282383
Mechanical Ventilation in the First Hour	12.502919939
Tracheostomy	8.840356153
Sex	7.161870971
Acute respiratory failure in the first hour	4.694937498
Systemic Arterial Hypertension	4.241766944
Non-invasive ventilation	3.962484574
Non-invasive ventilation in the first hour	2.846713391
Respiratory failure in the first hour	2.800651983
Morbid obesity	2.475677719
Non-invasive mechanical ventilation	2.319309057
Transfusion	2.189537788
Failure of non-invasive ventilation	1.466486287
Red blood cell concentrate	1.136906646

Table 3. Information gain per variable

Variable	Information gain
severe COPD	1.112180520
Complicated diabetes	1.052839037
Hypothyroidism	0.544533371
Delirium Obnubilation Stupor or Coma	0.525168608
Uncomplicated diabetes	0.389778122
Non-dialysis chronic renal failure	0.055503605
Psychiatric Illness	0.021600816
NYHA Classification 2 or 3	0.004103402
Length of stay in ICU	0.000000000
Lowest SBP 1h	0.000000000
Lowest PAD 1h	0.000000000
Lowest MAP 1h	0.000000000
Highest HR 1h	0.000000000
Highest FR 1h	0.000000000
Highest Temperature 1h	0.000000000
Higher leukocyte count 1h	0.000000000
Lower Platelet Count 1h	0.000000000
Higher PH 1h	0.000000000
Highest PaO2 1h	0.000000000
Higher PaCO21h	0.000000000
Highest Fi O21h	0.000000000
Higher PaO2Fi O21h Ratio	0.000000000

In Table 4, variables are shown that were eliminated when the area under the curve was less than 0.51.

Table 4. Area under the curve by variable

Variable	Area under the ROC curve
Duration of mechanical ventilation	0.7503002
Mechanical ventilation	0.6796403
Length of hospital stay	0.6544217
Urea	0.6337603
BUN	0.6337603
Central Venous Catheter	0.6317896
PAM catheter	0.6124523
Indwelling bladder catheter	0.6091883
Age	0.6064786
Mechanical ventilation	0.5923836
Highest creatinine 1h	0.5881882
Vasopressors	0.5875878
Vasopressors in the first hour	0.5695668
Mechanical Ventilation in the First Hour	0.5644553
Sex	0.5501601
Tracheostomy	0.5498214
Systemic Arterial Hypertension	0.5447869
Non-invasive ventilation	0.5330860
Morbid obesity	0.5302146
Non-invasive mechanical ventilation	0.5270969
Acute respiratory failure in the first hour	0.5258268
Non-invasive ventilation in the first hour	0.5175206
Transfusion	0.5171819
Failure of non-invasive ventilation	0.5166892
Uncomplicated diabetes	0.5138564
Complicated diabetes	0.5126016

Table 4. Area under the curve by variable

Variable	Area under the ROC curve
severe COPD	0.5124861
Hypothyroidism	0.5103461
Delirium Obnubilation Stupor or Coma	0.5092992
Red blood cell concentrate	0.5084370
Non-dialysis chronic renal failure	0.5024557
Psychiatric Illness	0.5020785
Respiratory failure in the first hour	0.5009161
NYHA Classification 2 or 3	0.5008699

Figure 1 presents the Pearson correlation analysis. Variables with a correlation greater than 0.75 were eliminated.

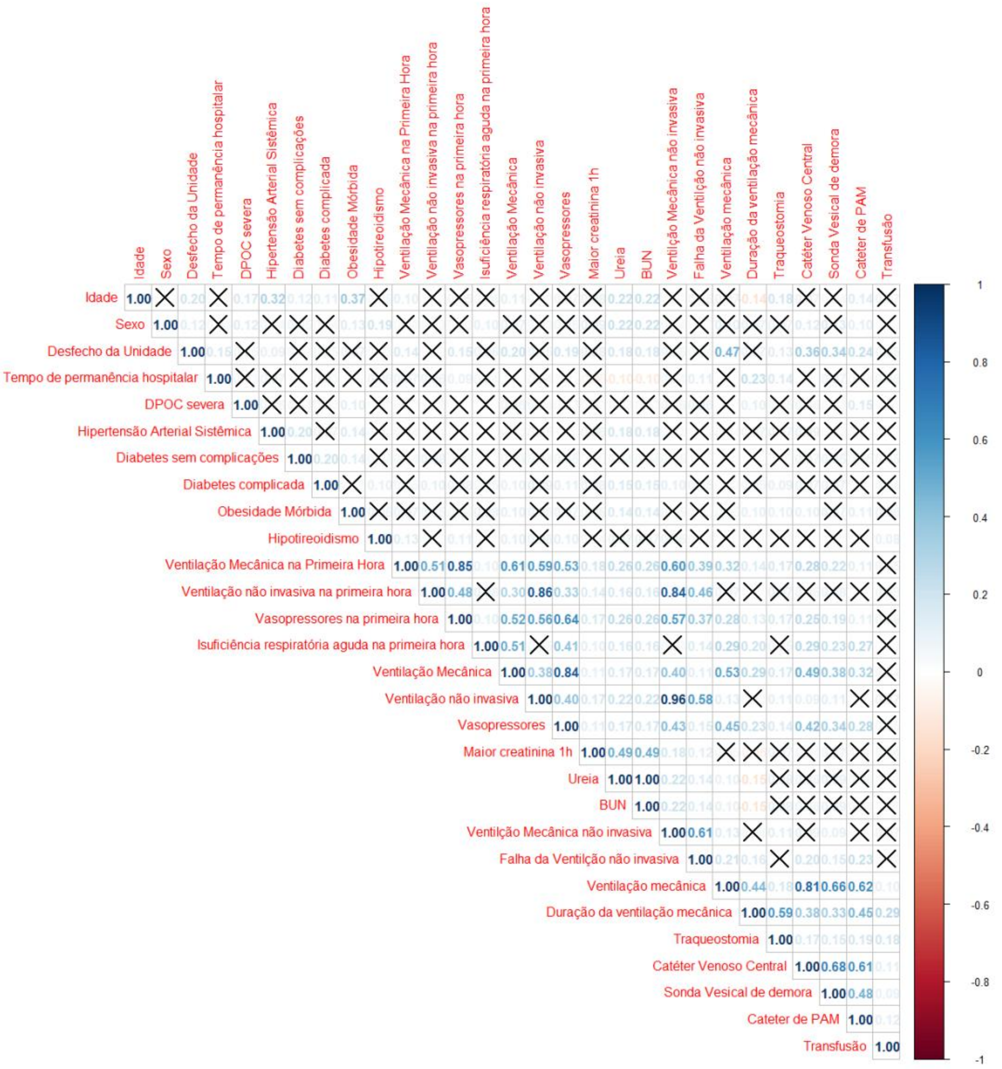

Association Rules

Association rules analysis is a technique for discovering how items are associated with each other (ZHAO; ZHANG; CAO, 2009).

The association rules were obtained by the apriori algorithm (ZHAO; ZHANG; CAO, 2009, p. 4) implemented by the arules library (HAHSLER, 2021) in R (TEAM, 2013). To use this algorithm it is necessary to convert the data set to a transaction database and the continuous variables need to be discretized, that is, dividing the values into intervals. Thus, age was divided into three categories: 0 to 55 years, 56 to 68 years and 69 to 95 years, length of hospital stay from 1 to 8, 9 to 17 and 18 to 522; highest 1h creatinine below 0.6, greater than or equal to 0.6 and less than 1 and from 1 to 28; BUN less than 21.5, greater than or equal to 21.5 and less than 34.7 and from 34.7 to 204; duration of mechanical ventilation

0 to 3, 4 to 11 and 12 to 60. The discretizations were made in order to have approximately the same number of samples in each class. Table 1 presents the first two transactions.

Table 1. Transaction database

## items ##	transactionID
[1] {Age=[56.69), ## Sex=F, ## Unit Outcome=Discharge, ## Length of hospital stay=[9.18), ## Severe COPD=False , ## Systemic Arterial Hypertension=True, ## Uncomplicated Diabetes=False, ## Complicated Diabetes=False, ## Morbid Obesity=True, ## Hypothyroidism=True, ## Non-invasive ventilation in the first hour=False, ## Vasopressors in the first hour=False, ## Acute respiratory failure in the first hour=False, ## Vasopressors=False, ## Highest creatinine 1h=[0.6,1), ## BUN=[0,21.5), ## Failure of Non-invasive ventilation=False, ## Duration of mechanical ventilation=[0.4), ## Tracheostomy=False, ## Central Venous Catheter=False, ## Indwelling Bladder Catheter=True, ## MAP Catheter=False, ## Transfusion=False}	1
## [2] {Age=[0.56), ## Sex=F, ## Unit Outcome=Discharge, ## Length of hospital stay=[18.522], ## Severe COPD=False, ## Arterial Hypertension Systemic=False, ## Uncomplicated diabetes=False, ## Complicated diabetes=True, ## Morbid obesity=False, ## Hypothyroidism=False, ## Non-invasive ventilation in the first hour=False, ## Vasopressors in the first hour= False, ## Acute respiratory failure in the first hour=True, ## Vasopressors=True, ## Highest creatinine 1h=[0.2,0.6), ## BUN=[0.21.5), ## Failure of non-invasive ventilation=False , ## Duration of mechanical ventilation=[12.60], ## Tracheostomy=False, ## Central Venous Catheter=True, ## Indwelling Bladder Catheter=True,	

```
## MAP Catheter=True,
## Transfusion=True}
```

two

Source: prepared by the author.

Mined association rules

The graph below represents the rules and their respective confidence levels, it can be generated interactively where we can hover the mouse and see the best rules (in this case the ideal ones based on confidence since we sort the data before creating the graph) . If it is interesting for your research, just let me know.

```
## Available control parameters (with default values):
## interactive = TRUE
## engine = htmlwidget
## max = 1000
## colors = c("#EE0000FF", "#EEEEEEFF")
## jitter = NA
## precision = 3
## main = Unused
## marker = list()
## verbose = FALSE
```

Figure 2. Association rules and trust level

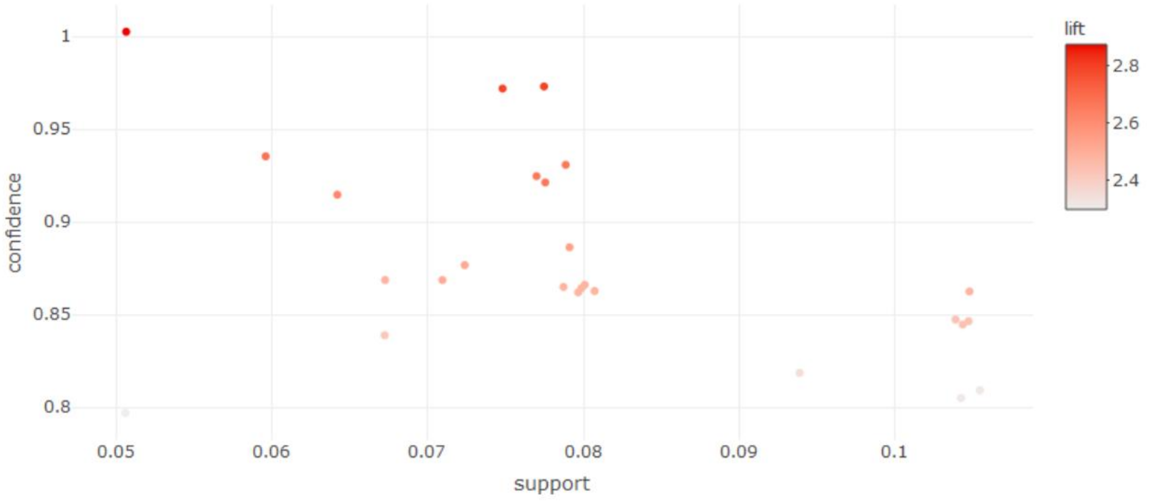

Table 5. Factors associated with the Discharge outcome

Lhs - factors	RHS - outcome	Support	Trust here	Covered frog	Lift	Quantity in
{Highest creatinine 1h=[0.6,1), Bladder catheter delay=False}	{Outcome of Unit=Discharge}	0.05031447	1.0000000	0.05031447	2.873494	24
{Indwelling Bladder Probe=False, Indwelling Catheter PAM=False}	{Outcome of Unit=Discharge}	0.07756813	0.9736842	0.07966457	2.797876	37
{Venous Catheter Central=False,Probe Bladder delay=False}	{Outcome of Unit=Discharge}	0.07547170	0.9729730	0.07756813	2.795832	36
{Arterial hypertension Systemic=True,Sound bladder delay=False}	{Outcome of Unit=Discharge}	0.05870021	0.9333333	0.06289308	2.681928	28
{Duration of mechanical ventilation=[0.4),Probe Bladder delay=False}	{Outcome of Unit=Discharge}	0.07966457	0.9268293	0.08595388	2.663238	38
{Vasopressors=False,Son bladder delay=False}	{Outcome of Unit=Discharge}	0.07756813	0.9250000	0.08385744	2.657982	37
{Vasopressors in the first hour=False, indwelling bladder catheter=False}	{Outcome of Unit=Discharge}	0.07756813	0.9250000	0.08385744	2.657982	37
{Highest creatinine 1h=[0.6,1),Central Venous Catheter=False}	{Outcome of Unit=Discharge}	0.06498952	0.9117647	0.07127883	2.619950	31
{Indwelling bladder catheter=False,Transfusion=False}	{Outcome of Unit=Discharge}	0.07966457	0.8837209	0.09014675	2.539367	38
{severe COPD=False,Probe Bladder delay=False}	{Outcome of Unit=Discharge}	0.07337526	0.8750000	0.08385744	2.514307	35
{Hypothyroidism=False, Sonda Bladder delay=False}	{Outcome of Unit=Discharge}	0.07127883	0.8717949	0.08176101	2.505097	34

Table 5. Factors associated with the Discharge outcome

Lhs - factors	RHS - outcome	Support	Trust here	Covered frog	Lift	Quantity in
{Obesity Morbid=False,Probe Bladder delay=False}	{Outcome of Unit=Discharge}	0.06708595	0.8648649	0.07756813	2.485184	32
{Indwelling bladder catheter=False}	{Outcome of Unit=Discharge}	0.07966457	0.8636364	0.09224319	2.481654	38
{Acute respiratory failure in the first hour=False, Indwelling urinary catheter=False}	{Outcome of Unit=Discharge}	0.07966457	0.8636364	0.09224319	2.481654	38
{Non-Invasive Ventilation Failure=False,Probe Bladder delay=False}	{Outcome of Unit=Discharge}	0.07966457	0.8636364	0.09224319	2.481654	38
{Tracheostomy=False,Son bladder delay=False}	{Outcome of Unit=Discharge}	0.07966457	0.8636364	0.09224319	2.481654	38
{Complicated diabetes=False,Probe Bladder delay=False}	{Outcome of Unit=Discharge}	0.07966457	0.8636364	0.09224319	2.481654	38
{Duration of mechanical ventilation=[0.4),Catheter Central Venous=False}	{Outcome of Unit=Discharge}	0.10482180	0.8620690	0.12159329	2.477150	50
{Venous Catheter Central=False, Catheter PAM=False}	{Outcome of Unit=Discharge}	0.10482180	0.8474576	0.12368973	2.435164	50
{Vasopressors=False,Caté have Central Venous=False}	{Outcome of Unit=Discharge}	0.10482180	0.8474576	0.12368973	2.435164	50
{Vasopressors in the first hour=False, Venous Catheter Central=False}	{Outcome of Unit=Discharge}	0.10482180	0.8474576	0.12368973	2.435164	50
{Non-invasive ventilation in the first hour=False, Venous Catheter Central=False}	{Outcome of Unit=Discharge}	0.06708595	0.8421053	0.07966457	2.419784	32

Table 5. Factors associated with the Discharge outcome

Lhs - factors	RHS - outcome	Support	Trust here	Covered frog	Lift	Quantity in
{Severe COPD=False,Catheter Central Venous=False}	{Outcome of Unit=Discha rge}	0.094339 62	0.81818 18	0.115303 98	2.3510 41	45
{Tracheostomy=False,Cat Central Venous Ether=False}	{Outcome of Unit=Discha rge}	0.104821 80	0.80645 16	0.129979 04	2.3173 34	50
{Venous Catheter Central=False,Transfusion= False}	{Outcome of Unit=Discha rge}	0.104821 80	0.80645 16	0.129979 04	2.3173 34	50
{BUN=[21.5,34.7),Central Venous Catheter=False}	{Outcome of Unit=Discha rge}	0.050314 47	0.80000 00	0.062893 08	2.2987 95	24

Source: prepared by the author.

Table 5 highlights sets of characteristics that are associated with patients' Discharge. For example, in rule 2 we can see that there is an association between a urinary bladder probe (false) and a MAP catheter (false) with Discharge. For this association rule we have a support of 0.077, which represents 37 (count) patients (7.7% of the total). The confidence column represents the confidence of this rule, where 0.9736 represents 97.36%%, that is, 97.36% of patients with a urinary bladder catheter (false) and MAP catheter (false) had Discharge. Furthermore, the lift (similar to odds) is 2.7978, this means that there is a 179.78% greater chance of having a Discharge.

Table 6. Factors associated with the unit outcome being Death

Lhs - factors	Rhs - outcome	Support It is	Trust nce	Cobert ura	Lift	Quantity ity
{Hospital length of stay=[9.18], Duration of mechanical ventilation=[12.60]}	{Outcome of Unit= Death}	0.10062 893	0.9795 918	0.10272 537	1,502 461	48
{Hospital length of stay=[9,18),Non-invasive Ventilation Failure=True}	{Outcome of Unit=D eath}	0.06289 308	0.9375 000	0.06708 595	1,437 902	30

**Table 6.** Factors associated with the unit outcome being Death						
**Lhs - factors**	**Rhs -<br>outcome**	**Support**<br>It is	**Trust<br>nce**	**Cobert<br>ura**	**Lift**	**Quantity<br>ity**
{Hospital length of stay=[9,18),BUN=[34.7,204]}	{Outcome of Unit=D<br>eath}	0.11320<br>755	0.9310<br>345	0.12159<br>329	1,427<br>985	54
{Hospital length of stay=[9.18],Highest creatinine 1h=[1.28]}	{Outcome of Unit=D<br>eath}	0.10062<br>893	0.9230<br>769	0.10901<br>468	1,415<br>780	48
{BUN=[21.5,34.7),Non-Invasive Ventilation Failure=True}	{Outcome of Unit=D<br>eath}	0.07337<br>526	0.8750<br>000	0.08385<br>744	1,342<br>042	35
{Hospital length of stay=[1.9),Duration of mechanical ventilation=[4.12)}	{Outcome of Unit=D<br>eath}	0.09853<br>249	0.8703<br>704	0.11320<br>755	1,334<br>941	47
{Age=[69.95],Duration of mechanical ventilation=[12.60]}	{Outcome of Unit=D<br>eath}	0.08176<br>101	0.8666<br>667	0.09433<br>962	1,329<br>260	39
{Hospital length of stay=[9,18), Catheter PAM=True}	{Outcome of Unit=D<br>eath}	0.23270<br>440	0.8604<br>651	0.27044<br>025	1,319<br>749	111
{BUN=[34.7,204],Duration of mechanical ventilation=[4,12)}	{Outcome of Unit=D<br>eath}	0.10062<br>893	0.8571<br>429	0.11740<br>042	1,314<br>653	48
{Uncomplicated diabetes=True,BUN=[34.7 ,204]}	{Outcome of Unit=D<br>eath}	0.11111<br>111	0.8548<br>387	0.12997<br>904	1,311<br>119	53
{Age=[69.95],Vasopressors=True deiro}	{Outcome of Unit=D<br>eath}	0.17190<br>776	0.8541<br>667	0.20125<br>786	1,310<br>088	82

Table 6. Factors associated with the unit outcome being Death

Lhs - factors	Rhs - outcome	Support It is	Trust nce	Cobert ura	Lift	Quantity ity
{Age=[69.95],Acute respiratory failure in the first hour=True}	{Outcome of Unit=D eath}	0.10691 824	0.8500 000	0.12578 616	1,303 698	51
{Hospital length of stay=[9,18), Venous Catheter Center=True}	{Outcome of Unit=D eath}	0.25995 807	0.8493 151	0.30607 966	1,302 647	124
{BUN=[21.5,34.7),Duration of mechanical ventilation=[4,12)}	{Outcome of Unit=D eath}	0.08176 101	0.8478 261	0.09643 606	1,300 363	39
{Age=[69.95],Vasopressors in the first hour=True}	{Outcome of Unit=D eath}	0.10482 180	0.8474 576	0.12368 973	1,299 798	50
{Hospital length of stay=[9.18], Vasopressors in the first hour=True}	{Outcome of Unit=D eath}	0.10482 180	0.8474 576	0.12368 973	1,299 798	50
{Age=[69.95],Duration of mechanical ventilation=[4.12)}	{Outcome of Unit=D eath}	0.09224 319	0.8461 538	0.10901 468	1,297 799	44
{Hospital length of stay=[9,18),Vasopressors=Green adeiro}	{Outcome of Unit=D eath}	0.17400 419	0.8383 838	0.20754 717	1,285 881	83
{Age=[69.95], Catheter PAM=True}	{Outcome of Unit=D eath}	0.19496 855	0.8378 378	0.23270 440	1,285 044	93
{Vasopressors=True,BUN=[34 .7,204]}	{Outcome of Unit=D eath}	0.20335 430	0.8362 069	0.24318 658	1,282 542	97

Table 6. Factors associated with the unit outcome being Death

Lhs - factors	Rhs - outcome	Support It is	Trust nce	Cobert ura	Lift	Quantity ity
{Hospital length of stay=[1,9),BUN=[34.7,204]}	{Outcome of Unit=Death}	0.10691824	0.8360656	0.12788260	1,282326	51
{BUN=[34.7,204],Tracheostomy=Fal only}	{Outcome of Unit=Death}	0.23270440	0.8345865	0.27882600	1,280057	111
{Severe COPD=True,Tracheostomy=Falso}	{Outcome of Unit=Death}	0.05241090	0.8333333	0.06289308	1,278135	25
{Vasopressors=False,Duration of mechanical ventilation=[4,12)}	{Outcome of Unit=Death}	0.08176101	0.8297872	0.09853249	1,272696	39
{Age=[69.95],BUN=[34.7.204]}	{Outcome of Unit=Death}	0.13207547	0.8289474	0.15932914	1,271408	63
{Vasopressors in the first hour=True,Tracheostomy=False O}	{Outcome of Unit=Death}	0.23270440	0.8283582	0.28092243	1,270504	111
{Hospital length of stay=[1.9],Highest creatinine 1h=[1.28]}	{Outcome of Unit=Death}	0.11111111	0.8281250	0.13417191	1,270147	53
{Age=[69.95],Venous Catheter Center=True}	{Outcome of Unit=Death}	0.24109015	0.8273381	0.29140461	1,268940	115
{Vasopressors in the first hour=True, PAM=False}	{Outcome of Unit=Death}	0.05870021	0.8235294	0.07127883	1,263098	28

Table 6. Factors associated with the unit outcome being Death

Lhs - factors	Rhs - outcome	Support It is	Trust nce	Cobert ura	Lift	Quantity ity
{Age=[69.95], Length of hospital stay=[1.9)}	{Outcome of Unit=D eath}	0.08805 031	0.8235 294	0.10691 824	1,263 098	42
{Vasopressors in the first hour=True, Duration of mechanical ventilation=[0.4)}	{Outcome of Unit=D eath}	0.05870 021	0.8235 294	0.07127 883	1,263 098	28
{Hospital length of stay=[9,18), Bladder catheter delay=True}	{Outcome of Unit=D eath}	0.27044 025	0.8216 561	0.32914 046	1,260 225	129
{Vasopressors in the first hour=True,BUN=[34.7,204]}	{Outcome of Unit=D eath}	0.15303 983	0.8202 247	0.18658 281	1,258 030	73
{Acute respiratory failure in the first hour=True,BUN=[34.7.204]}	{Outcome of Unit=D eath}	0.08595 388	0.8200 000	0.10482 180	1,257 685	41
{Hospital length of stay=[1.9), Venous Catheter Center=True}	{Outcome of Unit=D eath}	0.19916 143	0.8189 655	0.24318 658	1,256 098	95
{Hospital length of stay=[9,18), Duration of mechanical ventilation=[4,12)}	{Outcome of Unit=D eath}	0.13207 547	0.8181 818	0.16142 558	1,254 896	63
{Highest creatinine 1h=[1.28],BUN=[34.7.204]}	{Outcome of Unit=D eath}	0.20545 073	0.8166 667	0.25157 233	1,252 572	98
{Duration of mechanical ventilation=[12.60],Tracheostomy=Fal only}	{Outcome of Unit=D eath}	0.16352 201	0.8125 000	0.20125 786	1,246 182	78

Table 6. Factors associated with the unit outcome being Death

Lhs - factors	Rhs - outcome	Support It is	Trust nce	Cobert ura	Lift	Quantity ity
{BUN=[34.7,204],Non-Invasive Ventilation Failure=False}	{Outcome of Unit=Death}	0.23480084	0.8115942	0.28930818	1,244792	112
{Highest creatinine 1h=[1.28],Tracheostomy=False}	{Outcome of Unit=Death}	0.23480084	0.8115942	0.28930818	1,244792	112
{Hospital length of stay=[1.9),Vasopressors=True deiro}	{Outcome of Unit=Death}	0.15303983	0.8111111	0.18867925	1,244051	73
{Gender=M,Duration of mechanical ventilation=[4,12)}	{Outcome of Unit=Death}	0.15303983	0.8111111	0.18867925	1,244051	73
{Highest creatinine 1h=[1.28],Duration of mechanical ventilation=[4.12)}	{Outcome of Unit=Death}	0.09853249	0.8103448	0.12159329	1,242876	47
{Non-invasive ventilation in the first hour=False,BUN=[34.7,204]}	{Outcome of Unit=Death}	0.21383648	0.8095238	0.26415094	1,241617	102
{Age=[69.95],Uncomplicated diabetes=False}	{Outcome of Unit=Death}	0.17819706	0.8095238	0.22012579	1,241617	85
{BUN=[34.7,204],Central Venous Catheter=True}	{Outcome of Unit=Death}	0.24737945	0.8082192	0.30607966	1,239616	118
{BUN=[34.7,204],MAP Catheter=True}	{Outcome of Unit=Death}	0.21174004	0.8080000	0.26205451	1,239280	101

Table 6. Factors associated with the unit outcome being Death

**Lhs - factors**	**Rhs - outcome**	**Support** It is	**Trust nce**	**Cobert ura**	**Lift**	**Quantity ity**
{Vasopressors=True, Duration of mechanical ventilation=[0.4)}	{Outcome of Unit=D eath}	0.08805 031	0.8076 923	0.10901 468	1,238 808	42
{Sex=F,BUN=[34.7,204]}	{Outcome of Unit=D eath}	0.08805 031	0.8076 923	0.10901 468	1,238 808	42
{Sex=M, Vasopressors in the first hour=True}	{Outcome of Unit=D eath}	0.18238 994	0.8055 556	0.22641 509	1,235 531	87
{Hospital length of stay=[1.9), Bladder catheter delay=True}	{Outcome of Unit=D eath}	0.19916 143	0.8050 847	0.24737 945	1,234 808	95
{Hospital length of stay=[1,9), Catheter PAM=True}	{Outcome of Unit=D eath}	0.15513 627	0.8043 478	0.19287 212	1,233 678	74
{severe COPD=True,Probe Bladder delay=True}	{Outcome of Unit=D eath}	0.05031 447	0.8000 000	0.06289 308	1,227 010	24
{Complicated diabetes=True, Vasopressor s=True}	{Outcome of Unit=D eath}	0.05031 447	0.8000 000	0.06289 308	1,227 010	24
{Arterial hypertension Systemic=True,Complicated Diabetes=True}	{Outcome of Unit=D eath}	0.05031 447	0.8000 000	0.06289 308	1,227 010	24
{Complicated diabetes=True, Blood catheter PAM=True}	{Outcome of Unit=D eath}	0.05031 447	0.8000 000	0.06289 308	1,227 010	24

Table 6. Factors associated with the unit outcome being Death

Lhs - factors	Rhs - outcome	Support It is	Trust nce	Cobert ura	Lift	Quantity ity
{Systemic Arterial Hypertension=True,BUN=[34.7.20 4]}	{Outcome of Unit=Death}	0.21802935	0.8000000	0.27253669	1,227010	104
{Morbid Obesity=False,BUN=[34.7,204]}	{Outcome of Unit=Death}	0.22641509	0.8000000	0.28301887	1,227010	108
{Age=[69.95], Indwelling Bladder Probe=True}	{Outcome of Unit=Death}	0.25157233	0.8000000	0.31446541	1,227010	120

According to Table 6, the five most important variables for the Randon Forest model are time of use of mechanical ventilation, length of stay in hospital, age, BUN and highest 1h creatinine, however, the other variables, albeit on a smaller scale, contribute to explain the outcome.

Table 6. Cutoff point adjustment

Variable	Importance to the model
Duration of mechanical ventilation	51.3432130
Length of hospital stay	49.9806679
Age	19.6635518
BUN	16.6921254
Highest creatinine 1h	8.0331053
Central Venous Catheter = True	2.8502089
Indwelling bladder catheter = True	2.7456586
VasopressorsTrue	2.6986105
Acute respiratory failure in the first hour = True	2.3566367
Non-Invasive Ventilation Failure = True	2.3172256
Sex = M	2.0974426

Table 6. Cutoff point adjustment

Variable	Importance to the model
Vasopressors in the first hour = True	1.7933790
Uncomplicated diabetes = True	1.4712762
Tracheostomy = True	1.3493668
Hypothyroidism = True	1.3319429
Systemic Arterial Hypertension = True	1.2342152
Non-invasive ventilation in the first hour = True	1.1883745
Morbid Obesity = True	1.0033068
MAP Catheter = True	0.8561862
Transfusion = True	0.8297716
Complicated diabetes = True	0.6667737
Severe COPD = True	0.3312456

Waste analysis

As the residuals are randomized between -3 and 3, the model appears to be well adjusted and without outliers.

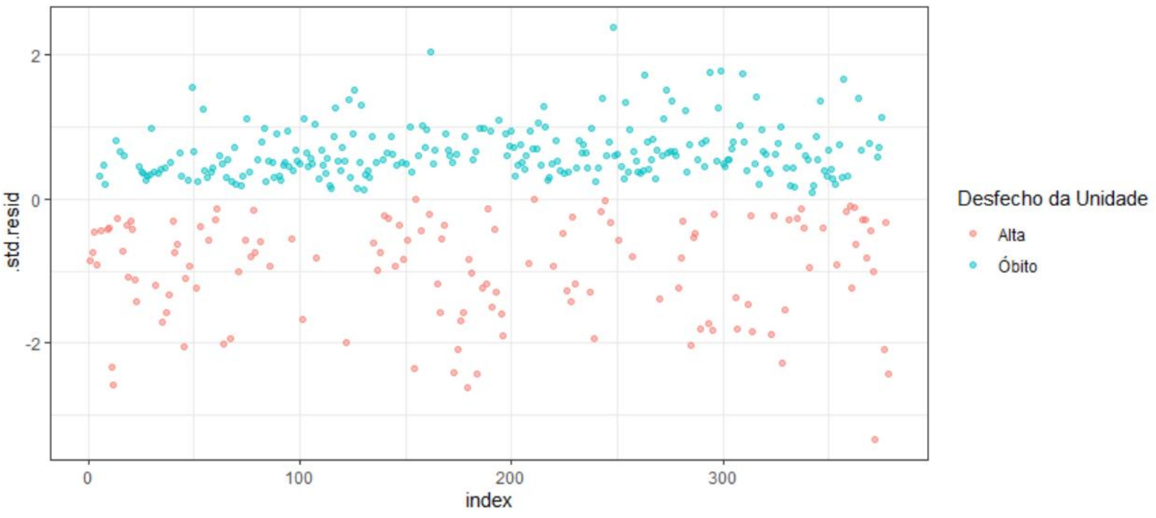

Figure 2. Standardized waste

The half-normal graph presented in Figure 1 attests to a good quality of adjustment of the logistic regression model to explain the Unit Outcome variable, since there are no points outside the envelope.

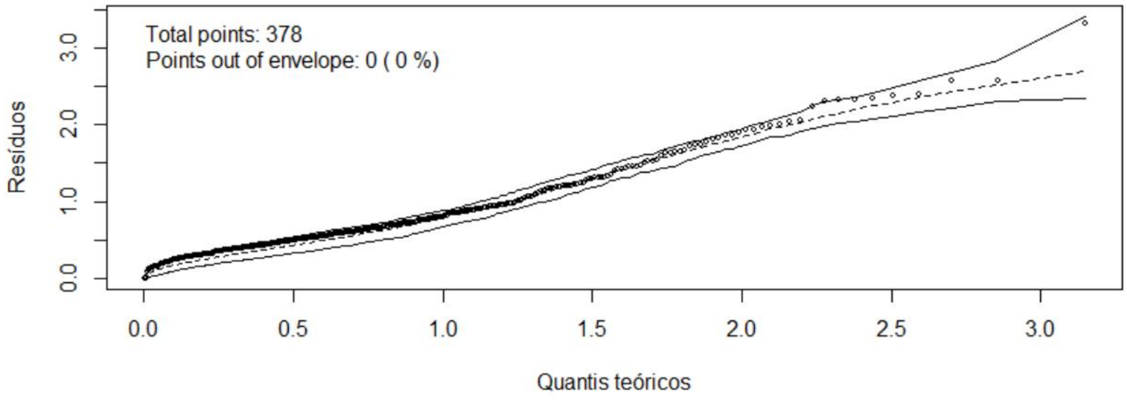

Figure 3. Half-normal graph and simulated envelope

References

GRAHAM, John W. Missing data analysis: Making it work in the real world. **Annual review of psychology,** v. 60, p. 549-576, 2009.

KUHN, Max. The caret package. **Journal of Statistical Software,** vol. 28, no. 5, 2009.

MEEYAI, Sutthipong. Logistic Regression with Missing Data: A Comparison of Handling Methods, and Effects of Percent Missing Values. **Journal of Traffic and Logistics Engineering,** vol. 4, no. 2, 2016.

MIDI, Habshah; SARKAR, Saroje Kumar; RANA, Sohel. Collinearity diagnostics of binary logistic regression model. **Journal of Interdisciplinary Mathematics,** vol. 13, no. 3, p. 253-267, 2010.

MORAL, Rafael A.; HINDE, John; DEMÉTRIO, Clarice GB. Half-normal plots and overdispersed models in R: the hnp package. **Journal of Statistical Software,** vol. 81, no. 1, p. 1-23, 2017.

PEDERSEN, Alma B. et al. Missing data and multiple imputation in clinical epidemiological research. **Clinical epidemiology,** vol. 9, p. 157, 2017.

PEPINSKY, Thomas B. A note on listwise deletion versus multiple imputation. **Political Analysis,** vol. 26, no. 4, p. 480-488, 2018.

SALMERÓN, Román; GARCÍA, CB; GARCÍA, J. Variance inflation factor and condition number in multiple linear regression. **Journal of Statistical Computation and Simulation,** v. 88, no. 12, p. 2365-2384, 2018.

TEAM, R. Core et al. **A: A language and environment for statistical computing.** 2013.

ZAWADZKI, Zygmunt; KOSINSKI, Marcin. **FSelectorRcpp:“Rcpp” Implementation of “FSelector” Entropy-Based Feature Selection Algorithms with a Sparse Matrix Support.** 2019. URL https://CRAN.R-project.org/package=FSelectorRcpp. R package version 0.3, v. 1, 2019.
