# Supplementary material for: Use of machine learning to identify protective factors for death from COVID-19 in the ICU: a retrospective study
Source: PeerJ. 2024 Jun 12;12:e17428. doi: 10.7717/peerj.17428 (PMC11179634; doi:10.7717/peerj.17428)
Supplement: Supplemental Information 3 [file peerj-12-17428-s003.doc]

STROBE Statement—Checklist of items that should be included in reports of ***cross-sectional studies***

|  | Item No | Information in the manuscript |
| --- | --- | --- |
| **Title and abstract** | 1 | Use of machine learning to identify protective factors for death from COVID-19 in the ICU |
| Introduction | | |
| Background/rationale | 2 | Patients in serious condition due to COVID-19 often require special care in intensive care units (ICUs). This disease has affected over 758 million people and resulted in 6.8 million deaths worldwide. Although the progression of the disease may vary from individual to individual, it is essential to identify the clinical parameters that indicate a good prognosis for the patient. Therefore, this research aimed to verify, through machine learning, the variables involved in the outcome of patients admitted to the intensive care unit due to COVID-19. |
| Objectives | 3 | the objective of this research was to verify through machine learning the variables involved in the outcome of patients admitted to the intensive care unit due to COVID-19. The results can help develop more effective preventive and therapeutic measures and patient care, providing better treatment and reducing the negative impact on public health. |
| Methods | | |
| Study design | 4 | This is an observational study with information from the medical records of patients admitted to the Intensive Care Unit due to COVID-19 in a Municipal Hospital in Paraná between March 2020 and July 2021 to verify the clinical aspects related to patient discharged |
| Setting | 5 | This study was divided into four stages: first) Data collection, second) filtering of variables to remove those that do not meet the criteria, third) selection of variables with the best performance to explain the outcome, and forth) identification of variables to estimate the risk for the outcome. |
| Participants | 6 | The study included all patients over 18 years of age admitted to the ICU of a public municipal hospital, a reference in the care of patients diagnosed with COVID-19, in the state of Paraná, southern region of Brazil. |
| Variables | 7 | One hundred twenty-six variables were collected from each patient and grouped into seven groups for a better understanding of the variable's nature. Variables: Age, gender, weight, height (cm); ICU stay duration, hospital stay duration, unit outcome (treatment/hospitalization outcome); Insuficiência renal crônica não dialítica, insuficiência renal crônica dialítica, cirrose child A ou B, cirrose child C, insuficiência hepática, tumor sólido locorregional, tumor sólido metastático, sítio do tumor, doença hematológica maligna, tipo de doença hematológica maligna, nome da doença hematológica maligna; Immunosuppression, severe COPD, AIDS, systemic arterial hypertension, asthma, uncomplicated diabetes, complicated diabetes, angina, previous myocardial infarction, arrhythmia, hypothyroidism, hyperthyroidism, peripheral arterial disease, chronic arterial fibrillation, rheumatic disease, sequelae of stroke, stroke without sequelae, dementia, smoking, alcoholism, psychiatric disease, morbid obesity, malnutrition, ischemic heart disease, dyslipidemia, history of pneumonia;  Delirium obnubilation stupor or coma, seizure or epilepsy, focal neurological deficit, cardiac rhythm disorders, respiratory failure in the first hour, cardiac arrhythmias in the first hour, cardiopulmonary arrest in the first hour, acute kidney injury in the first hour, asystole in the first hour, pulseless electrical activity in the first hour, atrial fibrillation in the first hour, sustained ventricular tachycardia in the first hour, acute respiratory failure in the first hour, arrhythmias, cardiopulmonary arrest, acute kidney injury, pulseless electrical activity, atrial fibrillation, sustained ventricular tachycardia, non-invasive mechanical ventilation, cardiac rhythm disorders, hypovolemic or hemorrhagic shock, septic shock, anaphylactic or undefined shock, BMI; Failure of non-invasive ventilation, duration of mechanical ventilation, tracheostomy, high-flow mask, duration of hemodialysis, extended hemodialysis in acute kidney injury, hemodialysis, asystole, non-invasive ventilation, vasopressors, hemodialysis in the first hour, mechanical ventilation in the first hour, non-invasive ventilation in the first hour, vasopressors in the first hour, decision on palliative care, chemotherapy, radiotherapy, solid organ transplant, autologous blood transfusion, intestinal transplant, lung transplant, kidney transplant, neurosurgery, central venous catheter, Foley catheter, MAP catheter, intra-aortic balloon, minimally invasive hemodynamic monitoring, red blood cell concentrate transfusion, fresh frozen plasma, thrombolytic agents, NYH classification 2 or 3, use of steroids; Lowest SBP 1h, lowest DBP 1h, lowest MAP 1h, highest HR 1h, highest RR 1h, highest Temperature 1h, lowest Glasgow Coma Scale 1h, highest Leukocyte Count 1h, lowest Platelet Count 1h, highest Creatinine 1h, highest Bilirubin 1h, highest pH 1h, lowest pH 1h, highest PaO2 1h, lowest PaO2 1h, highest PalCO 21h, lowest PaCO2 1h, highest FiO2 1h, lowest FiO2 1h, highest PaO2/FiO2 Ratio 1h, lowest PaO2/FiO2 Ratio 1h, highest Lactate 1h, Urea, BUN. |
| Data sources/ measurement | 8* | Patient information was collected using the Epimed Monitor ICU Database®, a platform where all clinical information of patients admitted to the ICU in Brazil is stored (21). Data came from electronic case report forms (eCRF) from which information is gathered through integration between electronic records (medical and/or administrative) of the hospital and manual entry of data made by a manager responsible for entering consecutively the information of each patient in the database. |
| Bias | 9 | The first is that it is an observational study in which the limits are due to the possibility that some essential variables have not been taken into account or the identification of risk variables. However, more than 126 variables were used here and were carefully selected to create a robust analysis model. The second limitation is that this is a study from a hospital in southern Brazil, which makes it challenging to make generalizations at a global level. However, as technical procedures are standardized across the national territory, and considering that Brazil has continental dimensions and the diversification of the Brazilian people, it is possible to infer that results can be obtained in other locations within the national territory. |
| Study size | 10 | 532 patients. |
| Quantitative variables | 11 | Described in Variables. |
| Statistical methods | 12 | Filtering variables with almost zero variance, missing values, lacking information, and information gain below 1 was performed. Sensitivity and specificity were used to assess the importance of each variable through the ROC curve, and those with values below 0.51 were eliminated. Additionally, variables with a correlation above 0.75, as determined by Pearson’s method, were excluded. We employed the decision tree technique and random forest to identify variables more sensitive to the outcome. Finally, logistic regression was applied to pinpoint variables associated with the patient’s discharge. Throughout the process of variable identification, VIF was used to test multicollinearity, and half-normal plot and simulated envelope techniques were employed. Logistic regression performance was assessed using sensitivity, specificity, and McNemar’s test. Statistical analyses were conducted using R software version 4.2, and results were considered statistically significant when p<0.05. |
| Results | | |
| Participants | 13* | 532 patients. |
| Descriptive data | 14* | According to Table 2, most patients were male (57.14%), without severe COPD (93.05%), and with arterial hypertension (70.11%). The majority, 66.92%, of patients did not have complicated diabetes, 92.29% did not have complicated diabetes, 79.51% were not morbidly obese, and 89.85% did not have hypothyroidism. Regarding patient care, most did not need non-invasive ventilation in the first hour (68.8%) or vasopressors (62.03%). However, 59.77% required vasopressors, 86.65% a central venous catheter, 90.41% a bladder catheter, and 73.87% an arterial catheter. Only 9.21% needed a transfusion. Regarding relative frequencies, the people who died presented one or more conditions: hypertension, need for vasopressors, central venous catheter, bladder catheter, and/or arterial catheter |
| Outcome data | 15* | Not performed. |
| Main results | 16 | According to Table 4, the chances of patients being discharged were 136% higher (OR 2.36; 95% CI, 1.28 - 4.44; p = 0.007) in females, 14% higher (OR 1.14; 95% CI, 1.10 to 1.19; p < 0.001) as hospital length stay increases, 737% higher when there was no central venous catheter (OR 8.37; 95% CI, 2.41 to 30.44 ; p < 0.001), 716% higher when there was no bladder catheter (OR 8.16; 95% CI, 2.30 to 32.14; p = 0.002). However, they decrease by 3% for each additional year of age (OR 1.97; 95% CI, 0.95 to 0.99; p = 0.011), and 9% smaller (OR 0.91; 95% CI, 0.86 to 0.96; p = 0.001) for each additional day of mechanical ventilation. |
| Other analyses | 17 | Table 5 presents the confusion matrix as a performance report of the variables used in logistic regression to verify the variables associated with the patient's discharge. Considering the training data, the logistic regression presents a balanced accuracy of 81.7%; that is, the model corrected 81% of the predictions, with a sensitivity of 74.6% regarding the ability to classify truly positive cases, and the specificity of 88.9% showing the ability to organize negative instances correctly. The test data's balanced accuracy was 85.5%, sensitivity 75.8%, and specificity 95.3%. The kappa value was 0.645, which indicates a substantial agreement between the predictions and the valid classifications. At the same time, the McNemar test showed no significant differences between the proportions of errors made by the models. |
| Discussion | | |
| Key results | 18 | This is the first study to collect metrics to determine associated factors that contributed to the death or discharge of these patients. Among the main factors, the variables, hospital length stay, central venous catheter, and bladder catheter, were related to the discharge of patients admitted to the ICU with COVID-19. In contrast, age and duration of mechanical ventilation were related to greater chances of going to death. The model's performance that detected these variables was satisfactory since the training and test data results are similar. |
| Limitations | 19 | This study has some limitations. The first is that it is an observational study in which the limits are due to the possibility that some essential variables have not been taken into account or the identification of risk variables. However, more than 126 variables were used here and were carefully selected to create a robust analysis model. The second limitation is that this is a study from a hospital in southern Brazil, which makes it challenging to make generalizations at a global level. However, as technical procedures are standardized across the national territory, and considering that Brazil has continental dimensions and the diversification of the Brazilian people, it is possible to infer that results can be obtained in other locations within the national territory. |
| Interpretation | 20 | Machine learning can identify patterns in the variables involved in the outcome of patients admitted to the intensive care unit due to COVID-19 and propose interventions and decision-making to improve epidemiological data and build care plans. Investigating and knowing the variables involved in the death and discharge of patients in serious situations are relevant metrics for improving treatment, diagnosis, and quality of care, reducing length of stay and mortality |
| Generalisability | 21 | Not applied because the study focused on Brazilian population. |
| Other information | | |
| Funding | 22 | We did not receive any external funding. However, as our research was conducted within a public Graduate Program in Brazil, it is mandatory to acknowledge the following information or funding in our acknowledgments: This study was partially financed by the Coordenação de Aperfeiçoamento de Pessoal de Nível Superior – Brasil (CAPES) – Finance Code 001.  This is necessary because directly or indirectly our program might receive somehow a funding. |
